# Human and environmental health concerns: What differences are required to make the difference?

**DOI:** 10.3389/fpsyg.2022.845388

**Published:** 2022-11-14

**Authors:** Joe Hinds

**Affiliations:** Psychology and Counselling, School of Human Sciences, University of Greenwich, London, United Kingdom

**Keywords:** environmental health, psychotherapy, unconscious, interdisciplinary, reflexivity

## Introduction

“*When your views on the world & your intellect are being challenged & you begin to feel uncomfortable because of a contradiction you've detected that is threatening your current model of the world...pay attention. You are about to learn something*”

(Drury, 1998, p. 201)

Taking an interdisciplinary perspective on the combined concerns of human and planetary health, this paper will turn to the subjectivity of psychotherapy as an important addition to the understanding and mitigation of psychological and environmental dis-ease. Psychology and psychotherapy, despite having a shared interest in people, generally exist at the opposite ends of a spectrum. Whereas, psychology perpetuates a more scientific and therefore a rather generic understanding of the human condition, psychotherapy attempts to deepen the understanding of the psyche at the individual and particular level. Both perspectives, alongside other important and relevant disciplines (e.g., Education: Bainbridge and Del Negro, 2020; Cybernetics: Bateson and Bateson, 2000), are required to address the environmental and health problems that humanity faces. Indeed, a reductionist undertaking, in any field, culminates in the creation of specialisms which restricts the holism required for adequate problem understanding and solving (Sennett, 2013). The central thesis of this paper is that there is a fostering of an inward, contemplative focus, alongside outward-focussed research, to develop some much-needed self-awareness. The “self” is understood in psychotherapy to be multiple (non-unitary), contextual, and dynamic, which if developed and explored may lead to “*moderating engrained responses and […] enhancing our relationships*” (Rose, 2012, p. 4; see also Schore, 2010) and potentially leading to more informed decision making.

Much of contemporary psychology and psychological research is inadequate for understanding (and therefore helping) the human condition (Sampson, 1981; Wertz, 1993; Notterman, 2000; Todres, 2003; Toomela, 2008; Dalal, 2018). As one example, there has been over 60 years of research focussed on the health implications of tobacco smoking (e.g., Doll and Hill, 1954). Despite this, it is estimated that tobacco will be responsible for 10 million deaths worldwide by 2025—a doubling of deaths in ~20 years (e.g., Proctor, 2004; World Health Organisation, 2021) making it the second leading global death risk factor (Ritchie and Roser, 2013). Moreover, the role of psychological interventions to prevent smoking has arguably been less than optimal (e.g., Shiffman, 1993; Schlam and Baker, 2013; Jackson et al., 2021). Essentially, affecting behavioral change in the last 60 years for such an important global health issue has been slow. In terms of contemporary psychological and environmental health issues—we simply do not have 60 years.

People don't typically change in any significant ways unless there is a strong personal motivation to do so or, regarding environmental crises, until it's too late (Diamond, 2004; SEI et al., 2021). People will die for their beliefs: Mike Hughes died crash-landing his self-built steam-powered rocket in an attempt to substantiate that the Earth was flat (Washington Post, 2020). In a similar myopic view, the HSBC plans to phase out coal mining financing by 2040 *but only* if it is cleared by shareholders (BBC, 2021).

Humans are far more than the information processing model espoused by the cognitive analogy derived from the advent of the computer in the 1950s (Beidel and Turner, 1986; Dreyfus and Dreyfus, 1986; Hardcastle, 1995). The human “processor” has far greater complexity and interconnectivity (depth) to it (e.g., Dijksterhuis et al., 2005). Knowledge about environmental issues (and indeed mental health), is by itself generally an “*insufficient precondition for successful action*” (Frick et al., 2004, p. 1598; see also Hicks and Bord, 2001; Kollmus and Agyeman, 2002; Loughland et al., 2003). For instance, a prominent theory within the social sciences regarding human behaviors, rational choice theory, merely reinforces the idea of what we *should* do but is “*fundamentally deficient as an account of* [actual] *behavior*” (Herrnstein, 1990, p. 356). Similarly, it has been suggested that psychology broaden its scope through multi-disciplinarity and to move beyond overly simplistic descriptions of “*events and causes*” (Belli et al., 2015, p. 762).

We can “know” that using fossil fuels, having a meat-based diet, working ourselves to an early breakdown, smoke, drink, choose less than suitable partners, etc. all have detrimental effects on our health, the environment (Oishi and Graham, 2010; Koger and Winter, 2011; Londakova et al., 2021) and our psyches (McWilliams, 2011; Jacobs, 2012)—yet we continue to do so. Why these behaviors have not attracted the same level of “pathologizing” that others have is a little confusing. With their obvious detrimental environmental and health related effects, these behaviors are not “*rational*.” They may be perceived, on the small scale as rather inconsequential (e.g., “it's only a short trip by car to the shops”; “what difference will one bacon sandwich make”; “I (only) have to produce more academic papers”) because they are “normal” and habitual. Take one example of a diagnosable condition—Internet Gaming Disorder (American Psychiatric Association, 2013): “*Compulsive, to the exclusion of other interests, and their persistent and recurrent … activity results in clinically significant impairment …People with this condition endanger their academic or job functioning [&] they experience symptoms of withdrawal*”. If we consider the criteria of this supposed condition, then why has there not been a Social Media Disorder, Alcohol Drinking Disorder or a Fossil Fuel Addiction Disorder? Furthermore, it has been proposed that if we are to follow a scientific objectivity and the subsequent diagnostic course, then we would have to include happiness as a disorder—*major affective disorder, pleasant type* (see Bentall, 1992, for a detailed account).

Individuals think little of their own behaviors because they are actively and largely unquestioningly engaging in many collective *irrational* behaviors daily—even when there is a rational “*knowing*” that they are detrimental. The uncomfortable conclusion here is that there may be a parallel between what is done normatively and those scientifically accepted diagnosable disorders of the DSM-5 (e.g., Granieri et al., 2017). Therefore, there is an increasing need to challenge the “*pathology of normalcy*” (Fromm, 1994, p. 27) or habit, or as phrased by Freud: *repetition compulsion* (Wilson and Malatesta, 1989) and to embrace a different mind-set, one that by its very nature of being “different” will disturb preconceptions. It may be worth taking, with reference to the opening quote, a reflexive account of your own responses and reactions to the presented material thus far.

The problem is that the human species tends to be change averse. This applies as much to you and your chosen academic community as it does to anyone observed in the population. We are just as likely to be dismissive of competing information than anyone else as a form of confirmation bias. Any engagement with alternative viewpoints could lead to uncomfortable dissonance. We are all therefore to some extent, employing various, and largely unconscious strategies, to avoid this uncomfortable state.

## The unconscious

“*Society has not yet been driven to seek treatment of its psychological disorders by psychological means because it has not achieved sufficient insight to appreciate the nature of its distress”*

(Bion, 1961, p. 14)

Ostensibly, psychodynamic theory and the concept of the unconscious is considered obsolete and outdated in much of mainstream psychology, albeit with a small and growing resurgence (Wilson, 2002; Bargh and Morsella, 2008; Bargh, 2014). Within this corpus are examples of research in cognitive, social, developmental and personality psychology, that now supports many Freudian-based propositions including that of the unconscious and various defense mechanisms that would need to be considered for “healing” (e.g., Baumeister et al., 1998; Westen, 1998). It has been said that “*most of our conscious thoughts are lies and fictions*” and that the start of the process of developing a sense of wholeness requires an active engagement with our unconscious (Fromm, 1994, p. 143). The term “healing” maybe defined as some degree of intra-personal “*wholeness*” (e.g., Powell, 2017).

Whilst there is a broad body of literature supporting the health and environment benefits of both social (e.g., Smith and Christakis, 2008) and environmental (inter-) connectedness (e.g., Cleary et al., 2017), there is less mainstream psychological interest about the importance of an integrated “self” (cf. Westen, 1992; Gigerenzer, 2007; Huta et al., 2012) and the role this could have in environmental and psychological well-being. However, the idea of an integrated (whole) self (cf. self-discrepancy theory: Higgins, 1987), forms a central feature of the act and art of psychotherapy (Jacobs, 2012). Bringing together the various disparate strands of the self (e.g., the unconscious into consciousness), and thus developing a greater depth and breadth of self-understanding, is its focus. The concept of the unconscious appears far less within mainstream health and environmental care literature (cf. Weintrobe, 2012).

It is the lesser-known parts of ourselves—the “shadow” selves (e.g., Bollas, 1987; Jung, 2014; Powell, 2018) that require greater attention if there is a genuine seriousness about eliciting change in the human condition. We will actively defend and justify the status quo of the system in which we operate because it largely prevents undue anxiety about the unpredictable nature of the world (Spinelli, 2007; Johnson and Fujita, 2012). The founder of rational emotive therapy Albert Ellis suggested that rational and conscious components of the psyche are more open to psychological interventions whereas the irrational and unconscious are “*subtle and tricky*” and less likely to be acknowledged or open to change: “*Practically all humans … are powerfully predisposed to unconsciously and habitually prolonging their mental dysfunction … they are obsessed with the pleasures of the moment rather than of the future, and that … is the main …. source of their resistance”* (Ellis, 1987, p. 365), and “*We are never trickier than when we are rationalionizing our resistances*” (Fromm, 1994, p. 115).

Human beings are adept at employing various, and largely unconscious strategies such as denial or suppression (see also *constriction of awareness*, Leigh and Reiser, 1982), that wishes the problem away or certainly our part in trying to solve or mitigate the problem. Or perhaps there is a tendency to maximize the importance of what we do (as academics) as a form of idealization (e.g., Trevithick, 2011), which then minimizes (de-values) other actions which could also be engaged in. Perhaps the state-of-affairs regarding both psychological and environmental health has not yet reached enough of a crisis for the pen to be given equal status with more directly relevant behaviors (unless these behaviors are perceived as someone else's responsibility?).

The profession of psychology (and others) promotes the important ability to debate a position from a critical perspective. However, this is rarely applied to our own embodied activities, beliefs and motivations. Without this *critical reflexivity* the defense of rationalization may develop, whereby we devise self-serving but incomplete explanations for our thoughts, emotions and behavior (e.g., Perry, 2014). An undefended testing of our own habitual positions in light of the knowledge we possess (Etherington, 2004; Finlay, 2008), allows a direct, experiential “knowing” and thus an empathic position about why others may also engage (or not) in important behavioral changes: we share greater similarities than differences. So alongside taking an authoritative position on the world through our choice of discipline, we spend some time developing an authoritative understanding of ourselves (Whitaker, 1976; Storr, 1999).

## What are the differences?

Psychotherapy is, or indeed should be a counter-cultural undertaking (e.g., Griffin, 2006); we simply cannot afford to either see or accept “things” as they purportedly are. Societal pressures of conformity, cultural beliefs, and norms are often the precursors of psychological and environmental dis-ease. Social and cultural consensus does not necessarily equate to being heathy (Searle, 2010; Powell, 2018): “*One demand made by our culture is like the demand of mother – we feel a symbiotic belongingness, addiction, & enslavement to the culture pattern”* (Whitaker, 1976, p.156). Perhaps our chosen disciplines, symbolize all-knowing parents.

To effect difference, we first need to be “different”; practitioners need to act as role models, to demonstrate their commitment to the problems that they face, by being genuine to ourselves and to those that we seek to help or influence. Role modeling has been applied to affect change in various ways (Clark et al., 2001; Oberg and Frank, 2009; Rizq, 2009; Sally, 2010) including within psychotherapy (Jacobs, 1989; Yalom, 2003). We cannot afford to perpetuate any sense of hypocrisy. If our primary driver as academics is about “*gleaning intellectual satisfaction from the work*” (Powell, 2018, p. 28), then we need to rethink our position. Moreover, scientists might “*abandon impact for insight*” (Belli et al., 2015, p. 762): It's important to be clever but it's more important to be useful.

If there is to be an understanding about the “oneness” described in this volume as a sense of inter-connectedness, then there needs to be consideration of a broad and inclusive sense of what exactly we are, or indeed need to be, connecting with. Whilst a strength has been developed in the more objective (and general) view of health and environmental problems, there is far more work to be done from the subjective position to understand our own intra-personal connections and contradictions. Despite all the advances and developments of mainstream psychology there remains an impoverished understanding of the human condition. A contemplative, critically reflexive approach maybe personally challenging and yet it is a vital twin to the established objective approaches harnessed to tackle the problems we face.

“*Trying to escape their inner struggles* & *contradictions they embraced unquestioningly one of …two sets of theories, blinding themselves to the merits of the other*”

(Bettelheim, 1960, p. 7)

## Author contributions

The author confirms being the sole contributor of this work and has approved it for publication.

## Conflict of interest

The author declares that the research was conducted in the absence of any commercial or financial relationships that could be construed as a potential conflict of interest.

## Publisher's note

All claims expressed in this article are solely those of the authors and do not necessarily represent those of their affiliated organizations, or those of the publisher, the editors and the reviewers. Any product that may be evaluated in this article, or claim that may be made by its manufacturer, is not guaranteed or endorsed by the publisher.
